# Epileptics and Responsibility

**Published:** 1905-04-22

**Authors:** 


### Hospital Clinics.

### EPILEPTICS AND RESPONSIBILITY.

Practical criminology, as far as its application is concerned, is a growth of recent years, for though so long ago as the early sixteenth century Sir Thomas More laid down some of its fundamental principles, he was too far in advance of his time for his thesis to gain acceptance. "If you allow your people," he says, "to be badly taught, their morals to be corrupted from childhood, and then when they are men punish them for the very crimes to which they have been trained in childhood, what is this but to make thieves and then to punish them?" But critical investigation in later years has made it abundantly clear that this wise attitude is far from comprehending the whole situation, for all men are not free agents to the extent accepted by public opinion and the law. The neuropathic inheritance has at its disposal so large a variety of interchangeable neuroses, that a just estimate of the correct scientific attitude towards criminals is attended with an extraordinary degree of difficulty.

This question of how far mental degeneracy is to be admitted as an excuse for crime, and the direction in which it should be allowed to modify the accepted relation between crime and its penalties, is likely to remain a vexed one for many generations of our successors, and it is easy to see the dangers attending too rapid a change of attitude in this great matter of personal responsibility. Already the line of cleavage is well-marked between the old school which is loth to accept the factor of inheritance, and that which gives the fullest possible recognition to inherent temperamental influences. For instance, Dr. T. H. Evans [1] has recently made two cases occurring in his experience the text of a plea for relieving criminals who are also epileptics from responsibility for their actions. The point of view, as representing that of an extreme "non-responsible," is worth consideration.

The first instance was that of a man, an undoubted epileptic, who committed murder. Though subject to epilepsy his attacks were not so frequent as to prevent his tenure, over long periods, of positions of a certain degree of trust. He was on one occasion dismissed from his situation, and was shortly after discovered in the act of pilfering the office of his late employer. The exact sequence of events is undetermined, but he certainly fell upon a watchman with a poker, and finally with a hatchet, and left him dead. The second case was that of an epileptic woman, who, in a fit of jealousy, stabbed her prospective husband in the heart. The wound happened to be not fatal, for the cavities of the heart were left intact, and suture of the injured myocardium resulted in recovery.

Dr. Evans's conclusions are as follows:—

1. "The essence of crime is in the intention, and the ability not so to intend." This axiom is scientifically just. The man who cannot check the impulse to gratify a grudge, or perpetrates an injury for which he has no motive whatever, is not a criminal, but a dangerous automaton, and, like all dangerous machinery, must be hedged about with safeguards. Where there is no crime it is idle to talk of punishment.

2. "No punishment is adequate to any crime; restraint, not only after a crime has been committed, but effort to hinder any such deed, is preferable always" (*sic*). Here, surely, is a dangerous heresy. In a sense, no doubt, it is true to say that no punishment is adequate to any crime, for one cannot scientifically express crime in terms of the punishment allotted to it. But though, as More said, the end of punishment is reformation, it certainly acts also in its deterrent aspect as a preventative, and to accept the proposition without reserve is to repudiate the experience of ages.

3. "The victims of epileptics ought to have legal ground for suit against the community as well as against those in charge of the epileptic." This is to say that, as a citizen who pays for police protection can claim against his community for compensation against mob violence, so should a sufferer at the hands of an epileptic be able to claim against his community for their improper control of an automaton. The plea seems to us altogether impracticable, for reasons which will appear.

4. "Reservations ought to be established in which degenerates and the morally irresponsible could be colonised and treated, allowing all possible freedom of initiative for useful and safe pursuits therein." This idealistic scheme glozes the fundamental obstacle—viz. the definition of "moral irresponsibility," and the means for its identification in time.

5. "Marriage of neurotics should be regulated." Again we ask, "What degree of neurosis is to be held a justification for restrictions?" Is the mild valetudinarian, who is distressed because his bowels are not relieved daily, to be included in the ban? Or the migrainous, the asthmatic, the sufferers from convulsive tics?

6. "All epileptics are to be viewed with suspicion." This is too general a proposition to be of any assistance. It is probably true that a man who has epilepsy is *ipso facto* less stable than his non-epileptic neighbours, but the degree of instability is subject to almost infinite gradation. Moreover, epileptic seizures sometimes occur only at intervals of years. Is the child who has had one seizure to be relegated thenceforth to an epileptic colony? We have given the author's conclusions in some detail, because they afford a good instance of the nebulous opinions which flock round all difficult problems. Nothing can be more mischievous, nor, we think, more false, than to say that all evil-doing is the evolution of heredity; such a creed is fatalism in its most repulsive shape, and is not warranted either by science or experience. Let it be granted that resistance to impulse is harder for some than for others; yet, except for the pure automaton, such as the automatic post-epileptic, a certain capacity for such resistance does exist in all men, and it is beyond all question that this capacity is stimulated by the deterrent penalties associated by law with the commission of crimes. Dr. Evans does not even claim for his epileptic murderers that they did what they did in the automatism of the post-epileptic state; it is enough that they were subject to epilepsy. No doubt it might justly be argued that the absence of an observed attack does not dismiss the possibility of its previous occurrence, but in each case there was a motive for the crime, and wherever there is a motive there is room also for the exercise of deterrents.

Destructive criticism is generally easy, but the matter of epilepsy and responsibility is too weighty to admit of ill-digested scheming. It is too much to hope that the marriage of neurotics can ever be regulated by legislative enactments. Such regulation can barely be achieved until public opinion is educated to the recognition of the stigmata of the neuropathic inheritance, to the gravity of marriage between neurotic individuals, and to the degree of self-sacrifice necessary for the discontinuance of such unfortunate unions. And by then the millennium will be upon us. At the same time it would seem that such education is our only means of protecting future generations, and, as such, deserves the constant support of the profession. Public opinion as at present constituted, which does not think it worth while to stamp out so preventable a scourge as syphilis, must alter out of recognition before it consents to regard the mental future of the race.

[1] Med. Rec., New York, Feb. 25, 1905.

